# The Impact of Service-Learning on the Prosocial and Professional Competencies in Undergraduate Physical Education Students and Its Effect on Fitness in Recipients

**DOI:** 10.3390/ijerph20206918

**Published:** 2023-10-13

**Authors:** Olalla García-Taibo, Isabel María Martín-López, Salvador Baena-Morales, José Eugenio Rodríguez-Fernández

**Affiliations:** 1 Department of Physical Activity and Sport Sciences, CESAG, Comillas Pontifical University, 07013 Palma de Mallorca, Spain; i.martin@cesag.org; 2 Department of General Didactics and Specific Didactics, Faculty of Education, University of Alicante, 03009 Alicante, Spain; salvador.baena@ua.es; 3 Department of Applied Didactics, Faculty of Education Sciences, University of Santiago de Compostela, 15782 Santiago de Compostela, Spain; geno.rodriguez@usc.es

**Keywords:** innovative methodologies, university education, physical activity, health, migrants

## Abstract

Education is a key component of the student’s transformation towards the creation of a more sustainable future. Among the methodological adaptations in teaching–learning processes, Service-Learning (SL) stands out as a meaningful academic experience to respond to social needs by developing committed citizens to transform society. The aim of the present study was to analyze the impact of this SL program on prosocial competence and satisfaction levels in university students, enhance physical fitness and analyze the reflections of the recipients. Moreover, the reflections on SL of the students and the migrants were analyzed. A mixed-methods design was performed. Forty-five students of Physical Activity and Sport Sciences provided a service to a migrant group that consisted of physical fitness training. The instruments implemented were the Prosocial and Civic Competence, the Impact of Service-Learning During Initial Training of Physical Activity and Sports and the reflective diary. The recipients participated in a physical fitness assessment and in a group discussion. The results show that SL in PAH contributes to pedagogical, communication, wellbeing and intercultural competences and also improves their prosocial and civic attitudes. Moreover, the recipients could enhance their physical fitness and their social interaction.

## 1. Introduction

Current education is undergoing a continuous change, which methodological advances in the teaching–learning process highlight. Among them, Service-Learning (SL) stands out as an active, responsive, experiential and educational model. In fact, it provides meaningful academic experiences in which students perform a service based on the needs identified in the community while improving and enhancing their understanding of the contents of the subject or course in which they are involved [1]. This innovative methodology enriches traditional learning environments by embedding theoretical concepts within real-world scenarios to drive community advancement [2]. Another fundamental aspect refers to the acquisition of constructive behaviors and the promotion of personal and social responsibility [3]. In other words, this pedagogical model is generally aligned with an educational system that attempts to respond to social needs and, therefore, develop citizens capable of getting involved and transforming their society.

Within academia, the enhancement of practical skills is crucial, requiring an interactive and participative learning approach involving the integration of values and diverse skills [4]. SL acts as a catalyst for individual and community transformation, aiming at holistic wellbeing. It enables students to impart knowledge and skills to service recipients in a practical way, concurrently refining their values and attitudes [5]. Consequently, SL provides optimal opportunities to bring the workplace closer to the educational context, consolidating the link between academic training and professional competencies. Accordingly, the inclusion of SL in the educational system is an opportunity for students to mobilize these general competencies prior to their incorporation into employment, guaranteeing an optimal adaptation to the tasks they will have to undertake [6]. The emphasis of this paper is on the pivotal role that education, specifically Service-Learning (SL), plays in the development of future physical educators and fitness professionals. This approach is instrumental in allowing individuals to make meaningful impacts within diverse communities, focusing on the transformative potential that physical educators can harness through practical experience with varied populations. It underscores the need for effective, community-centered education that fosters understanding and impacts our diverse society [7]. The aim is to emphasize the tangible impacts and practical skills acquired through SL, highlighting enhanced understanding and application in real-world contexts, particularly by future educators and fitness professionals.

In this context, SL experiences in higher education have increased in recent decades, showing several benefits for students [8]. SL is essential for student development, enabling the assimilation of essential life skills such as communication and collaboration and fostering a deeper understanding of societal issues [9]. Furthermore, exposure to tangible dilemmas allows students to apply theoretical knowledge more effectively, enhancing their overall understanding and approach to real-world problems [10]. Therefore, the comprehensive benefits of SL highlight its role in not just academic enhancement but also holistic personality development and growth [11]. By bridging the gap between academia and communities, SL contributes to achieving broader developmental goals and fosters an environment of critical thinking and innovative problem-solving [12]. Moreover, the incorporation of international SL programs introduces students to diverse global cultures, offering varying interactive learning landscapes and promoting a profound sense of civic responsibility and social consciousness [13]. On the other hand, it was recognized that existing Service-Learning frameworks have some limitations. There is a notable focus on practical elements related to a specific course. There is also a prominent emphasis on the institutionalization of SL, overshadowing mutual learning and academic outcomes. Furthermore, there is a significant research gap regarding the competent integration of Information and Communication Technologies (ICTs) and additional determinants that might influence the integration of ICTs in Service-Learning pedagogy. Consequently, it is argued that the technological perspective at different stages of SL has been neglected in current frameworks [14].

This extensive exploration of SL extends into numerous disciplines and finds notable applications in the fields of Physical Education (PE) and Physical Activity and Sport within higher education, setting the stage for further discussions on its implications in this domain [14]. In the PE context, previous studies have considered SL an option to accentuate authentic learning outcomes, critical reflection and civic engagement among university students [15,16,17]. Other benefits related to SL were obtained at the academic level (learning of contents, problem solving, attitudes towards learning or connection of knowledge with reality), personal development (change in thoughts, feelings, self-esteem and academic commitment), social interaction (ability to interact with others) and in the civic dimension (justice, honesty, etc.) [18]. Improvements have also been observed in cultural competence [19] and prosocial competence, especially in organizational skills, proactivity, tolerance and respect [20]. It is noteworthy that the reflective process promoted by this teaching model brings a dose of reality and awareness to students about their learning and contributions to the community [21]. Recently, a literature review concluded that this learning methodology presents excellent potential for development at the professional, personal and social levels, specifically in the PE student body. They highlight the existing connection between future PE professionals, the open and flexible characteristics of a continuously changing educational context and the reality of a diverse community. However, more SL studies in this area are needed [22]. As observed in the field of PE, numerous projects associated with SL have been developed. For instance, the development of university Service-Learning in physical activity and sports endeavors to pursue social inclusion (RIADIS, 2020) [23]. These programs exemplify the multifaceted approaches within SL to amalgamate academic learning with community service, thereby fostering an inclusive environment that promotes social integration through physical activity and sports. The incorporation of SL in such projects emphasizes the role of experiential learning in advancing inclusivity and social cohesion within the diverse context of PE.

In addition to all the above-mentioned positive aspects of SL for students, the benefits to the individuals receiving the service should be noted. Although some studies also focus on analyzing the benefits to community participants, most of the interventions that implement SL programs in the area of PE mainly focus on exploring the effects on students [22]. This type of program can contribute to the recipients in terms of education in values, improvement in coexistence in their society, satisfaction in the relationship with their teachers/coaches and healthy lifestyle changes [24]. Moreover, the implementation of SL through physical exercise programs allows for the transfer of all benefits related to physical activity (physical, psychological and social, among others) to the community [25,26].

Considering the potential of implementing SL in the academic area of PE, specifically through physical exercise programs to improve health, one of the main requirements is to find a curriculum link with the subject in which it will be integrated. In the areas of PE and physical activity, services based on sports training, recreation, rehabilitation and therapy, sports management and health promotion programs have been developed [27]. Regarding the studies on health promotion, the participants belonged to degrees related to health but not to PE. Since then, to our knowledge, no SL program has been developed within the Physical Activity and Health (PAH) subject of the Physical Activity and Sport Sciences degree (PASS). This course represents an ideal scenario to enhance healthy and autonomous habits in the community through physical exercise, while the students integrate and link the theory and practice of the subject. In relation to the general competencies associated with PAH, SL has the potential to contribute to many of them, such as the ability to organize and plan work, communication skills, problem solving, teamwork, ethical commitments, interpersonal skills, critical and self-critical capacity and the ability to learn, work autonomously or adapt to new situations, all of which are so appropriate to face today’s social diversity. As for specific competencies in PAH, SL is closely related to the ability to apply physical fitness assessment protocols, promote the acquisition of lasting and autonomous active habits, develop physical, behavioral and social principles, use sports material, equipment and spaces and practice the basic principles of training. In all the specific competencies of the subject mentioned above, the importance of being able to adapt their actions to different population sectors is emphasized.

As previously mentioned, most SL interventions are carried out in culturally diverse contexts, so that these experiences allow students to understand the social differences and cultural problems occurring in their community [18,28,29]. SL programs in PE include different conditions among the participants: people with special educational needs and people with other particular characteristics (victims of disasters, individuals of low socioeconomic status, specific ethnic groups), among others [16]. Among the vulnerable groups, or those at risk of social exclusion with severe economic difficulties, are migrants. As the Spanish Royal Academy of Language indicates, a person generally migrates for economic or social reasons. In Spain, many people migrate from Africa in pursuit of a better life, especially from Senegal. In the case of Mallorca, the social situation of people from Senegal involves significant integration difficulties. In this sense, migrants generally find themselves in a complicated situation since they neither understand nor speak the language, have a poor academic background and little professional experience. In addition, they find many barriers to accessing basic services, and, above all, the lack of document regularization results in a very high level of vulnerability and absence of protection, as it is an impediment to accessing the employment market [30]. For all these reasons, this collective was the recipient of the service provided by the students of the PASS degree in our study.

Therefore, the present study carried out an SL program with a group of students attending the PASS degree, specifically within the PAH course. The receptor group was a migrant group from Senegal living in Spain. In order to address the vulnerability of this collective and provide them with access to quality physical exercise, the main aim of this study was to analyze the impact of this SL program on Prosocial and Civic Competence and the satisfaction level of the students regarding SL. On the other hand, the aim was to evaluate the impact of SL on the physical fitness of the recipients, particularly on strength, cardiovascular endurance and body composition. Finally, the reflections about the SL experience of the students and the migrants were analyzed. We hypothesized that the recipients of SL would improve their physical fitness after the intervention. On the other hand, we hypothesized that the students engaging in SL would improve their Prosocial and Civic Competence and show high values in learning, pedagogical value, social impact and professional development.

## 2. Materials and Methods

### 2.1. Participants

The group comprised 45 PASS undergraduate students: 39 males (86.7%) and 6 females (13.3%), between 20 and 22 years old (average age = 20.8). The sample inclusion criterion was being enrolled in the PAH course, offered in the first semester of the 2020–2021 academic year. The group receiving the service was a group from Senegal living in Spain (*n* = 11; height: 180.0 ± 5.7 (cm); weight: 70.8 ± 10.2 (kg); BMI: 21.8 ± 2.5; % body fat: 16.1 ± 4.2; % muscle: 57.2 ± 7,9). An agreement was signed between the university and Caritas Mallorca, a Catholic humanitarian organization. After meeting with the director, a selection of migrants from Senegal was considered the most appropriate group for the implementation of the physical exercise service. The inclusion criteria for the recipients were: (i) being in a situation of vulnerability, (ii) being a migrant and (iii) passing the initial evaluation of health status for participation in a physical exercise program.

### 2.2. Instruments

The students completed the three following instruments: Prosocial and Civic Competence (PCC), Impact of Service-Learning During Initial Training of Physical Activity and Sports (IMAPS-AFD-FI) and the reflective diary. The PCC scale was used to analyze the promotion of prosocial attitudes through the implementation of Service-Learning projects in the field of PASS. This instrument was validated by expert judgment and showed very high Cronbach alpha (α = 0.89) [31]. The PCC consists of 31 items distributed in 6 dimensions: conformity to social correctness (3 items); social sensitivity (6 items); helpfulness and collaboration (4 items); safety and assertiveness in interaction (8 items); prosocial leadership (4 items); and social responsibility (6 items). The response options are presented on a Likert scale from 1 (strongly disagree) to 5 points (strongly agree).

The IMAPS-AFD-FI questionnaire was designed to observe the satisfaction level of the students when participating in SL projects. This questionnaire was validated to analyze SL experiences in the context of Physical Education by expert judgment, showing adequate factorial validity, an excellent Cronbach alpha (α = 0.95) and good values for each of the dimensions of the scale (α > 0.70) [32]. The instrument consists of 41 items distributed in 7 categories: identification context (9 open-ended questions); learning (5 items); pedagogical value (7 items); impact (6 items); professional development (4 items); professional competencies (7 items); and opinion (3 items and 1 open-ended question). Except for the first dimension and the final open-ended question, the rest of the items are answered using a 5-point Likert scale, with 1 being totally disagreeing and 5 being totally agreeing. The professional competencies factor was not included in the procedure. Also, given the quantitative nature of our research, the open-ended questions of the IMAPS-AFD-FI were not used in this study.

Finally, in relation to the reflective diary, as an instrument for individual deliberation, the students were invited to make a critical reflection and analyze and describe in detail the events or incidents that occurred during the intervention, their reactions and lessons learned for the future. The following open questions were transcribed and analyzed to better understand the competencies developed by students during and after the implementation of the SL experience: (a) a description of an event that occurred during the SL intervention through physical activity and movement games; (b) the reaction or response to the event; (c) determining the best response to the event; (d) the origin of the event; and (e) the ability to respond to future events based on knowledge of the event in question. The recipients of SL participated in a discussion group (see Appendix A for more detail).

In the “health status assessment”, the migrants completed the Par-Q test and a questionnaire for identification of cardiovascular risk factors (smoking, hypertension, cholesterol, family history, overweight, physical inactivity, glycemia) and signs/symptoms of diseases (cardiovascular, pulmonary, metabolic and musculoskeletal). Finally, the body composition monitor Tanita BC601 scale was used.

### 2.3. Procedure

This study follows a mixed-methods design, combining quantitative (uncontrolled before–after study) and qualitative (reflective diary and discussion group) research methodologies. It is part of a pre-competitive project of the CESAG (Universidad Pontificia de Comillas), entitled “Service-Learning Project: Being, leaving no one behind”, and was approved by the ethics committee of the university (code 2021/91). The service consisted of physical fitness training developed in the PAH subject. The SL activities were included in the regular planning of the course and, therefore, were mandatory for all students enrolled. Before the intervention, the student had been trained in the theoretical–practical content of the subject. Moreover, an introductory class was held on the characteristics of the SL methodology and the vulnerable situation of the migrants. The teacher in charge of the course continuously monitored the intervention.

**Figure 1:**
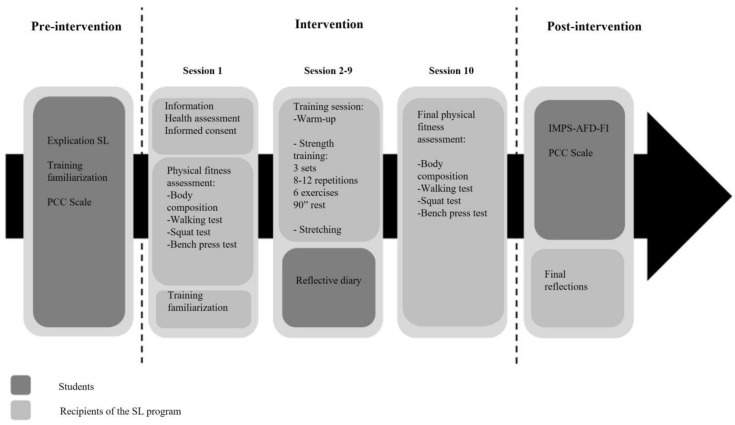
Description of the procedure.

The intervention with the service recipients consisted of 10 sessions of one and a half hours each, with a frequency of 1 session per week. On the one hand, all participants were informed of the details of the intervention in the first session. After this presentation, the recipients signed an informed consent form, and an initial assessment of the health status of each of them was performed. This was followed by an assessment of healthy physical condition and familiarization with the exercises included in the main training program. From the second to the ninth session, the training was carried out, consisting of a warm-up, a main part of strength training and a stretching phase. Finally, in session 10, the physical fitness assessment was repeated. On the other hand, as for the sample presenting the service, an initial assessment was performed using PCC. This was digitized and shared with the students through the university’s virtual platform. This assessment was repeated after the intervention was completed. In addition, after each training session, each student completed their reflective diary with their mobile device (see Figure 1), and they also completed one more questionnaire at the end of the intervention (IMAPS-AFD-FI). After the program, the migrants participated in a discussion group.

The PAH subject is mandatory and takes place over 15 weeks, with two theoretical classes (two and a half hours) and one practical class (one and a half hours) per week. The main objectives of the graduate curriculum to which the subject responded were: (i) to know and apply the most common measurement and instrumentation protocols; (ii) to promote lasting and autonomous habits of physical activity and sports practice among different population profiles; (iii) to apply the anatomical, physiological, biomechanical, behavioral and social principles in the different professional areas of physical activity and sport; (iv) to identify the health risks of inadequate physical activity practice; (v) to know how to select and use sports material and equipment; and (vi) to apply the basic principles of training.

The facility in which the service was performed consisted of a training classroom with gym equipment (machines, bars, weights, dumbbells, etc.) and a sports hall. These belong to the facilities of the university.

#### 2.3.1. Health Status Assessment

To determine the possibility of practicing physical exercise, an initial assessment of health status was performed. This consisted of the Par-Q test, identification of cardiovascular risk factors (smoking, hypertension, cholesterol, family history, overweight, physical inactivity, glycemia) and signs/symptoms of diseases (cardiovascular, pulmonary, metabolic and musculoskeletal).

#### 2.3.2. Physical Fitness Assessment

The walking test consisted of walking for 12 min at the maximum possible speed along a flat course. The exact distance covered was counted in meters, and VO2max was estimated using an equation [33].

For the estimation of 1-MR (one maximum repetition) in squat, as many repetitions as possible were completed with a submaximal load appropriate for the performance of 6–10 MRs (maximum repetitions). The load applied and the number of repetitions obtained were used to estimate the value of 1-MR [34].

The YMCA bench press test [35] consisted of repeating as many repetitions as possible in one minute while maintaining optimal exercise technique. The corresponding weight for men was 36 kg.

Body composition variables were also recorded with a Tanita BC601 scale (height, weight, BMI, percentage body fat and muscle).

#### 2.3.3. Strength Training

The strength training sessions consisted of 3 sets of 8–12 repetitions, with a 90 s rest between each exercise. Each set consisted of 6 exercises performed in the following order: biceps, bench press, half squat, triceps, deadlift and barbell rowing. Participants were encouraged to perform at least 8 and no more than 12 repetitions. If more than 12 repetitions were achieved in an exercise, the load was increased to remain in the prescribed intensity zone. The importance of controlling and assisting the exercise technique, including the breathing pattern, was emphasized, as were other aspects of safety and injury and/or accident prevention.

Before each strength training section, a warm-up of about 15 min was performed, which included 3 phases: general joint mobility, general activation (various displacements) and muscle activation, especially oriented to the groups involved in the main part of the session. Each training session ended with a 15 min stretching phase for the muscles involved.

### 2.4. Statistical Analysis

A descriptive analysis was performed for PCC (for each item and factor) and IMAPS-AFD-FI. With respect to the qualitative analysis, both for the final reflections of the migrants and for the reflective diaries of the students, open questions were transcribed and analyzed to better understand the experiences of both groups during and after the implementation of the SL experience. This analysis was carried out through a deductive–inductive approach. Thus, the evidence about the topic and the students’ narratives allowed us to establish the categories and quantify their frequencies. First, an open reading and coding of the reports were carried out, and then they were organized in relation to the theoretical framework. Finally, relevant quotations were selected to support the references in each category. Two researchers were involved in the analysis process and discussed their interpretations. A descriptive analysis of the studied variables for the fitness assessment was performed. The normality was verified using the Shapiro–Wilk test. Since the measures did not have a normal distribution and the sample was not representative, non-parametric tests were used. To answer the hypotheses, the Wilcoxon test was used, which made it possible to compare the differences between the pre- and post-intervention measures. Effect sizes were calculated as Cohen d, interpreted as small (0.2), moderate (0.5) or strong effect (0.8).

The statistical analysis of the data was performed with the IBM SPSS Statistic Program, version 29.0.1.0, and the level of statistical significance was set at *p*-value < 0.05.

## 3. Results

### 3.1. Effects of the Course on the Students

#### 3.1.1. Prosocial and Civic Competence—PCC Scale

Table 1 shows the comparison between the items of the PCC Scale before and after the intervention. There were significant differences in three items: Item 1: “I like to be generous with others and lend them my things if they need it” (*p* = 0.05; *d* = 31); Item 16: “When you talk to people and become intimate with them, you often discover values in them that you had not even suspected” (*p* < 0.05; *d* = 0.52); and Item 23: “I usually have no problem accepting and complying with the rules we follow in my house” (*p* < 0.05; *d* = 0.18). No significant differences were found in the rest of the items. However, most of them had higher scores after the intervention. The second hypothesis, which states that participation in SL improves PCC awareness among the students who provide the service, cannot be assumed.

A comparison between the dimensions of the PCC scale is displayed in Table 2. The dimensions with the highest scores both before and after the intervention were conformity to social correctness, social sensitivity and helpfulness and collaboration. No significant differences were found in any of the dimensions.

#### 3.1.2. Questionnaire on the Service-Learning Experience

With respect to the IMAPS-AFD-FI, all factors registered high scores, especially the pedagogical value (4.05) and the learning factor (3.92) (see Table 3).

#### 3.1.3. Reflective Diaries

This section presents the reflections extracted from the analysis of the 45 reflective diaries written by the students, structured into five categories: pedagogical competence, communication competence, wellbeing competence, intercultural competence and physical competence. Pedagogical competence was the dimension most referred to by students (37.68%) (see Table 4). The management of the training sessions (time organization, planification, collaborative work and content adaptation) and the ability to use appropriate methodologies were mentioned. The students declared an improvement in their capacity to better explain the exercises to the recipients. Some examples of their reflections were: “I realized that the recipient got frustrated when did not understand my explanation. Therefore, I know how to change the explanation of the exercise so that he could do it well without getting frustrated” (ID-17), and “When I realized that the recipient did not understand the explanation, I decided to describe the exercise step by step, from the smallest detail to the largest” (ID-25). Regarding the management of the training sessions, the students were aware of the importance of previously planning the sessions. Some examples of these reflections were: “I realized that we must structure warm-up and have the material prepared in advance” (ID-12), “I learned that it is necessary to dedicate time to plan the session before training a person” (ID-15), and “I learned that it is very important to make a previous explanation of everything we are going to do” (ID-26).

The next category more frequently mentioned by students was communication competence (24.64%), which referred to the abilities and knowledge to communicate with others. The students had trouble speaking in another language and realized that they could communicate with others through non-verbal communication, simplifying information and paying more attention to the recipients. Some examples of these reflections were: “I adapted my communication skills and simplify them to have an adequate and fluid communication” (ID-6), “Oral communication was difficult for us, since the recipient did not speak Spanish or English and we had to use gestural language” (ID-18), and “Although the communication was a bit complicated, we used signs or examples so that he could understand the technique of the exercise correctly” (ID-28).

Wellbeing competence was the next category with a greater number of references (15.94%). Students declared that experiences like SL are enriching both on a personal and a professional level. They also reflected on the importance of avoiding prejudices, being open-minded and offering the best version of themselves. Some comments that show personal and professional growth were: “I think SL is a very enriching process, since when the session evolves as expected, it gives us confidence for our future and growth as professionals” (ID-9), and “We are all the same, despite where we come from and whatever has happened to us. I believe that this experience will teach me to be a better person, and with that, I am satisfied” (ID-22).

The next category highlighted by students was intercultural competence (11.59%). This dimension referred to the ability to understand people of different social identities and their ability to interact with other cultures. The students pointed out their interest in a different culture and the activities, as well as the difficulties with the language for fluent communication. An example of their reflections was: “I learned a little more about the culture of their country and also improved my skills as a future graduate” (ID-38).

Lastly, physical competence was the category with a smaller number of references (10.14%). In this study, physical competence referred to the physical abilities (strength, aerobic fitness, flexibility, coordination) and the technique of the exercises performed. The students were aware of the importance of their physical abilities and knowledge of the technique of the exercises to facilitate the explanation and execution by the recipients. This can be observed in the following examples: “I realized how easy is teaching a trained person” (ID-40), “The problem was that no one had never explained him before how to warm up correctly and he had difficulties, since it was his first time” (ID-21), and “I learnt that the technique of an exercise is essential in order to perform an adequate movement” (ID-32).

### 3.2. Effect of the Intervention on the Recipients of the SL Program

#### 3.2.1. Fitness Assessment

Table 5 shows the descriptive analysis of the fitness assessment pre- and post-intervention. A significant improvement was observed after the intervention in the walking test (<0.05), squat test (<0.05), press bench (<0.05) and body mass index (BMI) (<0.05). No significant differences were found in the percentage of body fat and muscle. Therefore, the first hypothesis of our study, which states that the 10-week training intervention improves the fitness of the participants, is assumed.

#### 3.2.2. Final Reflections

In relation to the reflections recorded during the discussion group, all the participants expressed gratitude and were delighted to have carried out the program with the students. Regarding the benefits acquired, the majority reflected learning in relation to physical training: “before I didn’t know how to do it, and now I do know how to do it”, “I really liked it, learning to do the exercises well”, or “sometimes I did things in my training wrong, and now I already do them well”; some of them positively highlighted communication and social contact with other people: “I have loved talking with people, and learning how to do things” or “I liked meeting new people”.

## 4. Discussion

The aim of this study was focused on analyzing the effect of SL on two groups: on the one hand, the recipients of the service provided by the students, and on the other hand, the students of the Bachelor’s Degree in Physical Activity and Sport Sciences (PASS) who were taking the Physical Activity and Health (PAH) course.

In relation to the target group, a vulnerable group formed by adult African immigrants, this study concludes that after their participation in a physical conditioning program for 10 weeks, statistically significant differences were recorded in the evolution of their physical condition after this period of time. In addition to positively valuing the service provided, they highlighted the benefits at a social level (relationship with other people) and, specifically, the fact that students with adequate training conveyed the correct methodology to perform the exercises, both to increase their performance and to prevent possible injuries. It should be noted that the language barrier made it very difficult to obtain more detailed information about their experience during SL.

In [22], the authors highlighted the connection between Service-Learning (SL) and the contents of Physical Education, an open methodology that allows students to express their full potential. And, in this sense, the Degree in Physical Activity and Sport Sciences offers unbeatable training to students for professional development in different fields. Silva Piñeiro [36] points out the preparation and preference of students for the fields of Physical Education and Health, training and sports management, which are directly related to the project implemented in this study, an aspect that confirms the motivation of students for the design and development of this type of program. Regarding the improvement in the physical condition of the participants in the service, the application of a structured program of physical exercise causes these effects, obtaining greater benefits depending on the duration and intensity of the program, both in young adults [37] and in children and adolescents [38].

In relation to the students who designed and implemented the SL project and the PCC Scale on prosocial attitudes, the results of our study show that they obtained higher scores in all items after the intervention, registering statistically significant differences in “being generous with others” and “empathy”. Other studies [39,40] also emphasize the empathy of students when participating in SL projects due to their experiential nature and direct contact with the population, highlighting notable effects on the dimension of social sensitivity. Mesurado et al. [40] and Gil-Gómez et al. [31] highlighted the benefits in intrinsic motivation and in the sense of wellbeing that people experience when they dedicate their time to helping others, and, despite the fact that in certain cases the SL methodology is not associated with the wellbeing of the participants, causing a feeling of unhappiness [41], the students who implemented the project were indeed satisfied with the process and the outcome, showing high scores in the dimensions of social sensitivity and helpfulness and collaboration.

This feeling of wellbeing and happiness of the people participating in SL projects was also studied by [42], who, in an investigation on the effects of an SL program on 104 Physical Education students in the dimensions of subjective happiness, prosocial behavior and professional learning, recorded the positive impact on students in all dimensions, highlighting the positive effects of this methodology and its use at different educational levels and stages, considering it an optimal tool to address problems of social transformation [42,43]. Participating in this type of program with vulnerable groups provides students with in-depth knowledge of serious social problems that are a current reality in our country, and SL makes students more aware of the needs that are manifested in the recipients of the service [44].

As shown in Table 2 of our study, after the intervention in the SL program, all the items of the Prosocial and Civic Competence registered an increase compared to the pre-intervention values, except for “Safety and assertiveness in interaction”, which recorded practically no variation pre- and post-intervention. In this sense, it can be seen how SL, which has a high impact on the affective and social domains, helps to build more equitable societies [45] and promotes improvements in people’s prosocial behavior [46]. In relation to prosocial dimensions (social sensitivity, helpfulness and collaboration, safety and assertiveness in interaction, social responsibility and prosocial leadership), Owens et al. [47] point out that SL stands out for the aspects linked to humility and social equality (the highest values on the PCC scale in our study were related to social sensibility); Whitley et al. [48] affirms that participation in experiential programs such as SL causes notable improvements in the area of social responsibility (as reflected in our study, with an increase in this item from 3.57 to 3.70 after the SL intervention); and Sun et al. [49] highlight the development in prosocial leadership skills experienced by students after participating in SL (also contrasted in our study with an increase of 0.13 post-intervention).

In relation to the Impact of Service-Learning During Initial Training of Physical Activity and Sports (IMAPS-AFD-FI), our study recorded high scores in all factors (pedagogical value, learning, social impact and professional development), especially in the “pedagogical value” factor and the “learning” factor. Similar effects were recorded in the studies by González-Fernández et al. [46] in SL experiences with primary school students and by Chiva-Bartoll et al. [42], who, when analyzing the effects of an SL program on subjective happiness, prosocial behavior and professional learning based on the perceptions of Physical Education teachers training students, found positive effects in all dimensions of the IMAPS-AFD-FI, emphasizing the importance of the SL methodology in the development of professional competencies in students.

Capella-Peris et al. [43], Galvan et al. [50] and Whitley et al. [48] also highlighted the benefits of participating in SL projects in the learning and professional development of students, especially because of the direct contact with their social context and the deeper knowledge with diverse groups; and, in this sense, university students are the main beneficiaries of these experiences, who interrupt the more theoretical field of university classes and access a particular social context, interact with it and live practices and experiences that improve their academic and professional training.

Regarding the IMAPS-AFD-FI, our study recorded high scores in all factors (learning, pedagogical value, social impact and professional development), especially in the pedagogical value (4.05) and the learning factor (3.92). In this sense, Brown and Bright [51] stated that the “professional development” factor is closely linked to the “learning” factor, and this is developed through direct contact with a real work context. And a connection is also established between learning from the SL experience and the academic content of a given subject (otherwise, we would not be talking about SL). This positive impact of SL programs on the educational community as a whole, reflecting benefits at the academic, emotional, motivational and social responsibility levels of students, was also reflected in the study by González-Fernández et al. [46].

The information presented in the reflective diaries showed that a SL experience contributes to the development of and improvement in the skills required of PASS students. Communication skills, management of the training sessions, ability to understand people of different social identities and interact with them and intercultural abilities were the competences that the students in our study improved. Our findings reinforce the effectiveness of the SL methodology in improving personal and professional skills, in agreement with recent studies. Regarding developed competences, Hoolli et al. [52] concluded that SL is useful in the development of skills required in teachers. In the mentioned study, the subject involved in SL was English and not PAH; however, the development of competences was similar, highlighting the pedagogical and communication competences. In the same direction, Ruíz-Montero et al. [20] mentioned the opportunity of this type of experience to develop academic–professional competences, resilience, and management of different situations. Moreover, Ruíz-Montero et al. [20] underlined the development of emotional and personal learning, the improvement in empathy, a collaborative attitude and the ethical–civic commitment of students to society. Regarding intercultural competence, our findings reinforced that SL experiences could improve the capacity to develop an inclusive approach in Physical Education sessions [29]. In addition, the study of Chiva-Bartoll et al. [29], carried out with Physical Education teachers’ students, showed an improvement in their specific curricular subjects and an effective way of managing and promoting social cohesion and helping students develop their own identity. Therefore, this kind of experience may offer an empowering opportunity for PE students.

This study confirms that SL in the field of PE becomes a methodological strategy that combines theory, practice and community service in a single project, thus promoting the social dimension based on the SDGs analyzed by García-Rico et al. [26]. By linking education with the local environment and focusing on the integral development of the individual, SL in the field of PE is effectively aligned with the goals of SDG 4 (Quality Education) of the United Nations [53]. Therefore, PE transcends the teaching of motor skills to contribute to the formation of committed citizens and sustainable social development [54]. This approach reinforces the development of social and civic skills, encourages reflection on current problems in society and promotes, in the same way, inclusion and equality, aspects also pointed out in the studies of Chiva-Bartoll and Fernández-Rio [45] and Chiva-Bartoll et al. [55].

Regarding the strengths of this work, we would like to highlight the novelty of this manuscript, since, to our knowledge, this was the first SL program developed within the Physical Activity and Health subject of the Physical Activity and Sport Sciences degree. Moreover, compared to many studies that only included quantitative analyses, the design of this study used mixed methods, as proposed by Pérez-Ordás et al. [22]. However, we also found some limitations. We found it very difficult to motivate students to answer the different instruments included in our research with dedication, especially the diary, which had to be completed in each of the classes. Also, the language barrier of the migrant group made it difficult to obtain accurate and detailed information. With regard to the future line of research, we propose to improve the methodological quality by including a control group and a larger sample and to also try to simplify the data collection during the study in order to better engage students and obtain more valuable information.

## 5. Conclusions

The main goal of this study was to confirm the usefulness of the SL methodology in higher education, especially in PASS, in order to benefit not only the students but also the recipient group. Our results show that implementing SL in PAH is an opportunity to help students to contribute to their pedagogical, communication, wellbeing and intercultural competences and also to improve their prosocial and civic attitudes. Meanwhile, the recipients could improve their physical fitness and their social interaction. Therefore, this is a novel approach that contributes to a better understanding of the easy implementation of the SL methodology in the PASS degree in order to contribute to social transformation towards a more sustainable world.

## Figures and Tables

**Table 1:** Comparison between items of the PCC Scale pre- and post-intervention on students.

PCC Scale	Pre-Intervention		Post-Intervention		*p*-Value	*d*
	x	SD	x	SD		
Item 1	4.38	0.74	4.59	0.59	0.05	0.31
Item 2	4.29	0.76	4.60	0.58	0.10	0.46
Item 3	4.19	0.69	4.09	1.00	1.0	−0.12
Item 4	3.53	1.05	3.58	0.93	1.0	0.05
Item 5	3.89	1.20	3.83	1.17	0.32	−0.05
Item 6	2.00	1.13	2.28	0.98	0.14	0.26
Item 7	4.47	0.73	4.47	0.73	1.0	−0
Item 8	4.03	0.76	4.12	0.65	0.48	0.13
Item 9	3.84	1.05	4.04	0.81	0.71	0.21
Item 10	3.50	0.79	3.69	0.79	0.16	0.24
Item 11	3.84	0.90	4.28	0.61	0.07	0.57
Item 12	3.45	0.74	3.56	0.77	0.16	0.15
Item 13	3.74	0.86	3.84	0.80	0.18	0.12
Item 14	4.24	0.61	4.15	0.54	0.32	−0.16
Item 15	3.81	0.65	3.96	0.79	0.26	0.21
Item 16	3.86	0.92	4.25	0.53	0.03	0.52
Item 17	4.14	0.74	3.96	0.79	0.10	−0.23
Item 18	4.03	0.83	4.00	0.80	1.0	−0.04
Item 19	4.00	0.89	4.04	0.66	0.56	0.5
Item 20	3.50	0.97	3.35	0.85	0.71	−0.16
Item 21	4.17	0.85	4.20	0.76	0.08	0.04
Item 22	2.96	1.07	3.12	1.03	0.74	0.15
Item 23	4.04	1.00	4.20	0.71	0.03	0.18
Item 24	4.43	0.63	4.65	0.49	0.06	0.39
Item 25	4.48	0.72	4.62	0.57	0.13	0.22
Item 26	4.53	0.73	4.73	0.45	0.13	−0.36
Item 27	3.86	0.79	4.27	0.72	0.10	0.73
Item 28	4.00	0.96	3.96	0.84	0.13	−0.04
Item 29	3.94	1.03	3.96	1.04	0.41	0.02
Item 30	4.68	0.68	4.52	0.71	0.41	−0.23
Item 31	4.24	0.61	4.15	0.54	0.32	−0.06

Note: x = average; SD = standard deviation; *p*-value was established by 0.05 of significance, and Wilcoxon test was used.

**Table 2:** Comparison of the dimensions of the PCC scale between dimensions pre- and post-intervention.

Dimensions of the PCC Scale	Pre-Intervention		Post-Intervention		*p*-Value	*d*
	x	DS	x	DS		
Conformity to social correctness	4.25	0.81	4.34	0.72	0.39	0.12
Social sensitivity	4.18	0.80	4.36	0.64	0.39	0.25
Helpfulness and collaboration	4.12	0.82	4.21	0.65	0.41	0.12
Safety and assertiveness in interaction	3.95	0.85	3.92	0.83	0.45	−0.04
Social responsibility	3.57	0.90	3.70	0.84	0.28	0.15
Prosocial leadership	3.70	0.85	3.87	0.75	0.13	0.21

Note: x = average; SD = standard deviation; *p*-value was established by 0.05 of significance, and Wilcoxon test was used.

**Table 3:** Statistical description of the satisfaction of SL among students (*n* = 39).

IMAPS-AFD-FI	x	SD
Learning	3.92	0.87
Pedagogical value	4.05	1.12
Social impact	3.76	0.95
Professional development	3.58	0.99

Note: x = average; SD = standard deviation.

**Table 4:** Number of references in each category of students’ reflective diaries (*n* = 45).

Categories	No. of References (%)
Pedagogical competence	107 (34.29%)
Communication competence	75 (24.04%)
Wellbeing competence	67 (21.47%)
Intercultural competence	40 (12.82%)
Physical competence	19 (6.09%)

**Table 5:** Descriptive analysis of the effect of the course on the physical fitness of the recipients (*n* = 7).

	Pre-Intervention		Post-Intervention		*p*-Value	*d*
	x	SD	x	SD		
Weight	70.84	10.19	67.79	5.55	0.02	−0.37
BMI	21.85	2.51	21.26	1.31	0.03	−0.29
% Body fat	16.13	4.18	16.86	3.2	0.35	-
% Muscle	57.25	7.94	55.49	6.34	0.73	-
Walking test	1610.04	143.93	1689.79	128.19	0.03	0.58
Squat test	89.22	14.89	106.25	22.17	0.01	0.90
Bench press test	23.63	11.94	30.86	11.65	0.02	0.61

## Data Availability

The data presented in this study are available on reasonable request from the corresponding author.

## Appendix A

Questions asked to the migrant group to record their final reflections after the SL intervention:

Question 1: What do you think of the fact that university students came to do physical activity and exercises with you?

Question 2: How do you think you have benefited the most?

Question 3: Did you like the exercises proposed?

Question 4: Please be honest and if there are things you didn’t like, you can also say so.

Question 5: Do you find it useful to do physical activity together with the students? Why?

Question 6: Do you think that your physical condition (strength, flexibility, endurance and agility) has improved after these weeks of physical practice with the students?

Question 7: Do you notice that you relate more and/or better with others?

Question 8: Do you think that, when interacting with students and people different from your everyday environment, your way of thinking or reflecting may be different?

Question 9: How did you feel or what did you think when you were doing physical activity with students?
